# Impact of Serine/Threonine Protein Kinases on the Regulation of Sporulation in *Bacillus subtilis*

**DOI:** 10.3389/fmicb.2016.00568

**Published:** 2016-04-20

**Authors:** Frédérique Pompeo, Elodie Foulquier, Anne Galinier

**Affiliations:** Laboratoire de Chimie Bactérienne, CNRS, UMR 7283, Institut de Microbiologie de la Méditerranée, Aix-Marseille UniversitéMarseille, France

**Keywords:** Ser/Thr protein kinases, phosphorylation, regulation, sporulation, *Bacillus subtilis*

## Abstract

Bacteria possess many kinases that catalyze phosphorylation of proteins on diverse amino acids including arginine, cysteine, histidine, aspartate, serine, threonine, and tyrosine. These protein kinases regulate different physiological processes in response to environmental modifications. For example, in response to nutritional stresses, the Gram-positive bacterium *Bacillus subtilis* can differentiate into an endospore; the initiation of sporulation is controlled by the master regulator Spo0A, which is activated by phosphorylation. Spo0A phosphorylation is carried out by a multi-component phosphorelay system. These phosphorylation events on histidine and aspartate residues are labile, highly dynamic and permit a temporal control of the sporulation initiation decision. More recently, another kind of phosphorylation, more stable yet still dynamic, on serine or threonine residues, was proposed to play a role in spore maintenance and spore revival. Kinases that perform these phosphorylation events mainly belong to the Hanks family and could regulate spore dormancy and spore germination. The aim of this mini review is to focus on the regulation of sporulation in *B. subtilis* by these serine and threonine phosphorylation events and the kinases catalyzing them.

## Introduction

**FIGURE 1:**
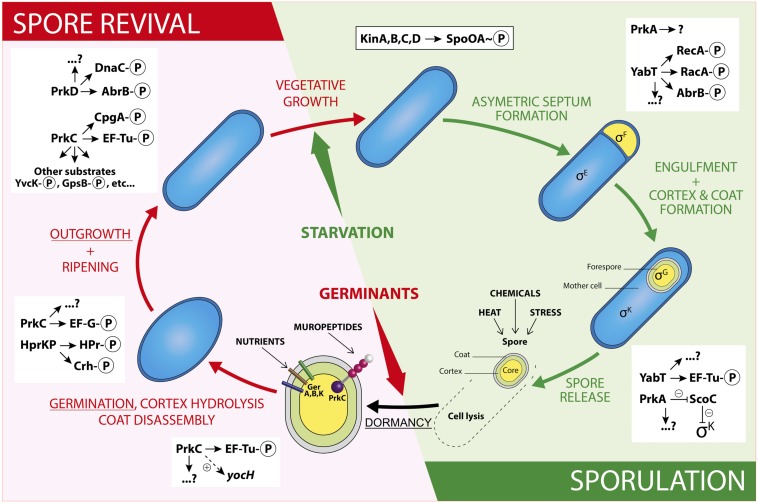
**Schematic illustration of regulations by Ser/Thr phosphorylation during a *Bacillus subtilis* spore life.** The sporulation steps are presented in the green panel and the spore revival steps in the red panel. The stages described in the review are underlined. In the schematic spore, the core is in yellow, the cortex in green and the coat in gray. The two types of spore signal receptors are represented: the GerA, B, and K in sticks, the STPK PrkC in balls (purple: kinase domain, pink: PASTA domains, and white: IgG-like domain) and stick (transmembrane domain). For a detailed structure of a STPK, see (Pereira et al., 2011). For examples of PrkC substrates identified during vegetative growth, see references (Foulquier et al., 2014; Pompeo et al., 2015). The two-component cascade leading to Spo0A phosphorylation is presented in the framed rectangle and regulations by STPKs are listed in the white rectangles. Other possible cross-regulations by two-component systems, BY-kinases, or other phosphorylations are not represented here in order to not overload the drawing.

Many Gram-positive bacteria form endospores in response to stress or nutrient limitation (Stragier and Losick, 1996; Higgins and Dworkin, 2012). Spores are morphologically distinct cells that are highly resistant to heat, chemicals, and radiation (Nicholson et al., 2000; Setlow, 2003, 2006, 2008). These dormant cells are able to reinitiate growth rapidly in response to environmental signals like amino acids or cell-wall muropeptides released by growing cells (Setlow, 2003, 2008). These processes are well regulated and orchestrated by a series of protein phosphorylation events and changes in gene expressions controlled by sigma factors (σ^E^, σ^F^, σ^G^ and σ^K^). In *Bacillus subtilis*, a landmark of the initiation of sporulation is the activation of the transcriptional master regulator Spo0A. It is activated by phosphorylation through a remarkable multi-component phosphorelay system of autophosphorylating histidine kinases (KinA-KinE) (Burbulys et al., 1991; LeDeaux et al., 1995; Tan and Ramamurthi, 2014). These phosphorylations are labile and highly dynamic, thus permitting a temporal regulation of the sporulation initiation decision (De Jong et al., 2010). More recently, another kind of phosphorylation, more stable yet still dynamic, on serine or threonine residues of protein substrates, has been proposed to play a role in sporulation (Cousin et al., 2013). These phosphorylation reactions are generally catalyzed by Ser/Thr protein kinases (STPKs) of the Hanks family (Hanks and Hunter, 1995). They seem to regulate entry into sporulation, dormancy, and spore revival. These kinases share a common fold for their cytosolic catalytic domain typically composed of 12 subdomains organized in two-lobes surrounding the active site (Kornev and Taylor, 2010). In some STPKs, the kinase domain is attached to a transmembrane helix connected to an extracellular ligand binding domain responsible for kinase activation. In some, the kinase domain is connected to a transmembrane helix without any extracellular domain. In others, the kinase domain is soluble. They are themselves activated by autophosphorylation on Ser or Thr residues of their activation loop (Pereira et al., 2011). In addition, each kinase is able to phosphorylate various substrates on Ser and/or Thr residues. In *B. subtilis*, four Ser/Thr kinases of the Hanks family have been characterized to date: PrkA, PrkC, PrkD, and YabT. All of them, except PrkD, are implicated at different levels of the sporulation process: onset, dormancy, germination, and outgrowth (**Figure 1**) (Shah et al., 2008; Bidnenko et al., 2013; Yan et al., 2015). Several phosphoproteome studies have been performed during the last 10 years and thanks to technical progress especially in mass spectrometry, more and more phosphorylated proteins have been identified in *B. subtilis* (Eymann et al., 2007; Macek et al., 2007; Soufi et al., 2010; Kobir et al., 2011; Ravikumar et al., 2014; Rosenberg et al., 2015). In early studies, only one experimental condition was probed. Nowadays, dynamic phosphoproteomes can be analyzed, and allow to explore several growth conditions for the same bacterial population. Furthermore, it has recently been proposed that cross-talks exist between two-component systems and STPKs as well as cross-phosphorylations among STPKs (Pereira et al., 2011; Shi et al., 2014a). This network of regulations may also be complicated by cross-talk with bacterial tyrosine kinases (BY-kinases) (Cousin et al., 2013). Such a complex regulatory network could allow quick and efficient regulation of bacterial physiology in response to the environmental variations. Broadly, the role of phosphorylation in several bacterial processes like DNA-related mechanisms, cell division and morphogenesis have been discussed recently (Garcia-Garcia et al., 2016; Manuse et al., 2016). In this review, we will specially focus on regulations mediated by STPKs during the different stages of sporulation in the model bacterium *B. subtilis*.

## Sporulation

**FIGURE 2:**
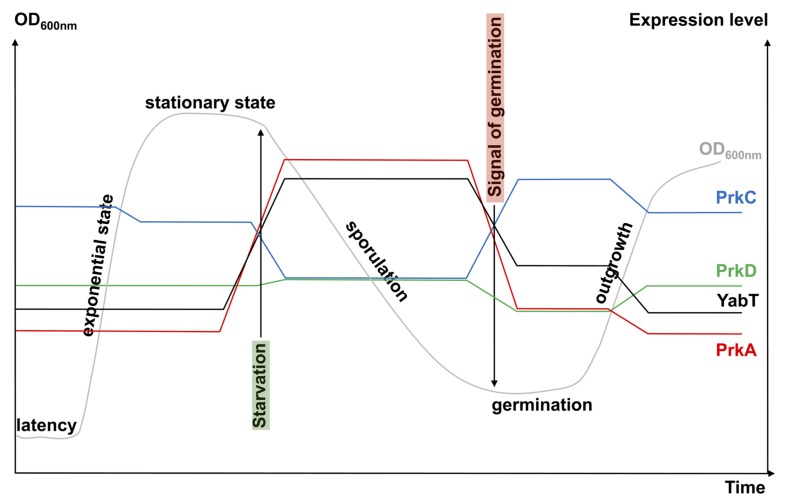
**Schematic view of STPK gene expression during growth.** The growth curve of a *B. subtilis* cell culture in rich medium is presented in gray (OD_600nm_). The levels of kinase gene expression are obtained from http://subtiwiki.uni-goettingen.de/ (Michna et al., 2016) and from Nicolas et al. (2012). They are presented in color: blue for *prkC*, green for *prkD*, red for *prkA*, and black for *yabT*. Signals for starvation (highlighted in green) and germination (highlighted in red) are indicated by arrows.

Sporulation is a morphological differentiation event that is initiated by an asymmetric division yielding a smaller forespore and a larger mother cell (Stragier and Losick, 1996; Higgins and Dworkin, 2012). Several steps are necessary from the formation of a forespore to the release of a mature spore after lysis of the mother cell. These include engulfment, cortex synthesis and coat formation (**Figure 1**) in order to confer to the spore the resistance properties required to survive extreme conditions of temperature, desiccation and ionization (Setlow, 2006). This robustness is the result of several factors like dehydration, DNA compaction, and dormant metabolism (Nicholson et al., 2000; Setlow, 2007; Camp and Losick, 2009; Doan et al., 2009). As mentioned before, initiation of sporulation is controlled by a cascade of phosphorylation events catalyzed by two-component systems and eventually leading to the activation of Spo0A. When the level of Spo0A-P is sufficient (Vishnoi et al., 2013), the compartment-specific transcription factor σ^F^ is activated, definitely engaging the sporulating cell into a specific program of genes expression (Tan and Ramamurthi, 2014). In addition, the expression of two genes encoding the STPKs PrkA and YabT increases strongly during sporulation under the control of the spore-specific sigma factors, σ^E^ and σ^F^, respectively (**Figure 2**). These two kinases have been indeed shown to participate in the regulation of several mechanisms occurring during the initiation of sporulation (Fischer et al., 1996; Bidnenko et al., 2013; Yan et al., 2015). PrkA is a STPK that only possesses a catalytical domain and localizes in the coat of the forespore (Eichenberger et al., 2003). This protein also shows a distant homology to eukaryotic cAMP-dependent protein kinases and several essential residues of their active site are apparently conserved in PrkA. Using a *B. subtilis* crude extract, it has been proposed that PrkA phosphorylates an unidentified 60-kDa protein on Ser residue(s) (Fischer et al., 1996). However, no PrkA autophosphorylation was detected. That is surprising since STPKs generally need to be autophosphorylated to be active. However, even if the enzymatic properties of PrkA are poorly characterized, the role of this protein in sporulation seems clearly established. Actually, deletion of *prkA* gene leads to a sporulation defect corresponding to a delay in the entry into sporulation and a decrease in the number of spores. It has recently been shown that PrkA was involved in the synthesis of the σ^K^ transcription factor (Yan et al., 2015). Indeed, PrkA increases the expression of σ^K^ and its downstream target genes, by inhibiting the negative transcriptional regulator ScoC (Hpr) (**Figure 1**). However, the complete mechanism of regulation, potentially via the kinase activity of PrkA, is not known: how does PrkA act on ScoC? Is it a direct or indirect regulation of ScoC and does PrkA phosphorylate ScoC on Ser/Thr residue(s)? What are the exact targets of PrkA phosphorylation? Though it appears that PrkA is a key player in the regulation of sporulation in *B. subtilis*, more work needs to be done in order to completely understand the role of this putative STPK. The second regulatory protein YabT is a STPK containing three domains: a transmembrane region, a kinase domain and a DNA-binding domain. YabT kinase activity has been clearly established and targets of YabT have been identified. Binding of YabT to DNA activates its kinase activity; YabT is then able to autophosphorylate and to phosphorylate exogenous substrates (Bidnenko et al., 2013). It colocalizes with the septal inner membrane separating the forespore from the mother cell. As for *prkA*, deletion of the *yabT* gene leads to a sporulation delay. Furthermore, resistance to DNA damages decreases in the *yabT* mutant spores. Both phenotypes were also observed in a *recA* mutant (Shafikhani et al., 2004; Bidnenko et al., 2013). The recombinase RecA is actually a YabT substrate *in vitro* (phosphorylation on Ser2) and consistently, RecA was previously identified in a phosphoproteome study revealing the same phosphorylated residue (Soufi et al., 2010). It is, therefore, likely that YabT regulates RecA activity in the forespore in order to allow DNA damage repair before nucleoid compaction in the spore (Sciochetti et al., 2001). Similarities between the bacterial STPK YabT and the eukaryotic STPKs C-Abl and Mec1 have been found: all these kinases are activated by DNA and phosphorylate proteins involved in DNA damage repair mechanisms (Bidnenko et al., 2013). *In vitro*, YabT is able to phosphorylate RacA, another DNA-related protein involved in DNA anchoring to the cell pole (Ben-Yehuda et al., 2003; Shi et al., 2014b). RacA can be dephosphorylated *in vitro* by SpoIIE, a serine protein phosphatase known to modulate the phosphorylation state of the anti-anti σ^F^ factor SpoIIAA (Arigoni et al., 1996). Interestingly, YabT and SpoIIE have been found associated to the same protein partners in a recent yeast two-hybrid screen suggesting that they function as a kinase/phosphatase couple during sporulation (Shi et al., 2014b). Another potential substrate of YabT is the global transcriptional regulator AbrB which is phosphorylated on Ser86 *in vivo* (**Figure 1**) (Soufi et al., 2010) as well as *in vitro* by YabT, PrkC and PrkD (Kobir et al., 2014). AbrB is a global gene regulator involved in transition phases (i.e., from exponential to stationary growth phase) that also antagonizes sporulation by repressing the expression of Spo0A (Phillips and Strauch, 2002). It has been proposed that AbrB phosphorylation serves as an additional input for a subtle control of AbrB activity. Indeed, AbrB phosphorylation inhibits its ability to bind its DNA targets (Kobir et al., 2014). In addition, a strain expressing phospho-mimetic AbrB produces fewer spores and sporulates much slower. Because the YabT kinase is produced just after the onset of sporulation, it is the best candidate for AbrB phosphorylation in sporulation conditions.

## Dormancy

It is commonly accepted that when the mature spore is released by the mother cell, it is metabolically dormant and environmentally resistant. The spore is protected by thick layers: the cortex and the coat, and contains a high level of dipicolinic acid (DPA) and a low amount of water. However, it has been recently shown that the spore RNA profile is highly dynamic a few days following sporulation (Segev et al., 2012). During this short period, spores are responsive to environmental changes and can adapt their RNA content consequently. Furthermore, some enzymatic activities necessary for full maturation of coat proteins have been described in spores (Zilhão et al., 2005; Sanchez-Salas et al., 2011). Taking these observations into account, it is possible to consider that regulation of enzymatic activities or protein synthesis by phosphorylation reactions exist in spore during this adaptive period. For example, the overall metabolism is down regulated, in particular protein synthesis, which is an energy-intensive cellular process. This regulation is mediated by phosphorylation of the elongation factor Tu (EF-Tu). Indeed, phosphorylated EF-Tu is unable to hydrolyze GTP and remains bound to the ribosome which leads to a dominant-negative effect in elongation, thus inhibiting protein synthesis (Pereira et al., 2015). It has been proposed that the kinase involved in this phosphorylation is YabT because it is present in the spore during dormancy and it is able to phosphorylate EF-Tu *in vitro* on Thr63. *In vivo* experiments confirmed that YabT is responsible of EF-Tu phosphorylation in the spore since no phosphorylated EF-Tu was found in a *yabT* mutant (**Figure 1**) (Pereira et al., 2015). Moreover, *in vitro* phosphorylation of EF-Tu by PrkC was previously reported on Thr384 but the *in vivo* regulatory function of this phosphorylation has not been accounted for so far (Absalon et al., 2009).

## Germination and Outgrowth

Spores of *B. subtilis* can remain dormant for years but return to life quickly after exposure to nutrients or muropeptides (Setlow, 2003, 2008, 2014). Specific receptors (including GerA, GerB, and GerK) that detect nutrients have been known for years (Atluri et al., 2006; Ramirez-Peralta et al., 2013). More recently, in the inner spore membrane, the STPK PrkC has been shown to bind muropeptides released by growing cells thus inducing germination (Shah et al., 2008). Spores can also reinitiate growth stochastically at a low frequency due to phenotypic variations in individual spore (Sturm and Dworkin, 2015). The revival process (**Figure 1**) can be divided into three consecutive phases: (i) germination with spore rehydration, release of DPA, cortex hydrolysis, and coat disassembly, then (ii) a ripening period with no morphological changes but a molecular reorganization of the cell, and finally (iii) outgrowth with synthesis of macromolecules, membrane elongation, and cell division (Sinai et al., 2015). However, it seems that synthesis of proteins might start earlier, as early as 30 min after the initiation of germination. Up to 650 new proteins are synthesized during the three steps described above (Sinai et al., 2015). A dynamic phosphoproteome of reviving spores established a functional connection between Ser/Thr/Tyr-phosphorylation and progression of this process (Rosenberg et al., 2015). Though it was proposed that the STPK PrkC, and, therefore, phosphorylation of PrkC protein substrates, stimulates germination only in the presence of muropeptides as germinant (Shah et al., 2008), this phosphoproteome analysis was only done in the presence of L-Ala as germinant. It will be interesting to perform the same study using muropeptides as germinant to compare the profile of phosphorylated proteins identified. Nevertheless, the high number of new phosphoproteins already characterized (Rosenberg et al., 2015) suggests an important modulation of protein activity during this cellular transition to vegetative growth. The phosphoproteins identified are involved in spore-specific functions, transcription, metabolism, and stress response, some of which are probably phosphorylated by STPKs. YabT and PrkA are highly synthesized during sporulation whereas PrkC is more expressed during germination (**Figure 2**). However, these three kinases and possibly other still unknown protein kinases (with weak homology to classical STPK) could contribute to these regulatory mechanisms. PrkC is a transmembrane protein composed of an intracellular catalytic domain and an extracellular regulatory C-terminal region containing several beta-lactam-binding domains. These PASTA domains (for penicillin-binding protein and serine/threonine kinase-associated domains) are predicted to interact with the peptidoglycan (PG) (Yeats et al., 2002). Biochemical studies of PrkC homologues confirmed the *in vitro* interaction between PG fragments and PrkC (Mir et al., 2011; Ruggiero et al., 2011; Squeglia et al., 2011). Thus, binding of PG fragments released from growing cells to the extracellular domain of PrkC could stimulate PrkC kinase activity to induce the germination of the spore (**Figure 1**) (Shah et al., 2008). The *prkC* gene expression is low during sporulation and stimulated during germination but its expression level during vegetative growth and especially during stationary phase is not negligible (**Figure 2**). Hence, PrkC can phosphorylate several substrates produced during vegetative growth or sporulation and, up to now, more than 10 substrates of PrkC have been identified *in vitro*. These targets include proteins of carbon metabolism (Pietack et al., 2010) or proteins involved in protein synthesis like CpgA, a GTPase involved in a late stage of ribosome assembly, and the elongation factors EF-G and EF-Tu (Shah et al., 2008; Absalon et al., 2009; Pompeo et al., 2012). It has beeen proposed that PrkC phosphorylates EF-G in the spore to allow re-initiation of protein synthesis. But, it is unlikely that this phosphorylation is the only cause of germination. PrkC also promotes the expression of *yocH*, a muralytic enzyme encoding gene. YocH is exported and digests the PG of other growing bacteria, thus producing more muropeptides that in turn stimulate germination (Shah and Dworkin, 2010; Libby et al., 2015). Moreover, the phosphorylation of several proteins has been shown to be important for germination. These include the spore specific proteins SspA et SspB involved in DNA protection, the ribosomal protein RpsJ, the elongation factors EF-Tu and EF-G, and the phosphocarrier protein HPr (Rosenberg et al., 2015). But, for many of them, the kinase that catalyzes their phosphorylation is still unknown. In the particular case of HPr, which is a component of the phosphoenolpyruvate-dependent sugar system (PTS) and a key player of carbon catabolite regulation in *B. subtilis*, phosphorylation is catalyzed by HprK/P, an atypical ATP-dependent kinase/phosphorylase. This enzyme does not share homology with eukaryotic STPKs and does not belong to the Hanks kinase family. Instead, it shares limited homology with the phosphoenolpyruvate carboxykinase (Galinier et al., 2002). It does not autophosphorylate but phosphorylates two protein substrates, HPr and its homologue Crh, on the Ser46 residue (Galinier et al., 1997, 1998). HprK/P is stimulated by phosphorylated sugars like glucose 6-phosphate or fructose 1,6-bisphosphate (Jault et al., 2000) for the regulation of carbohydrate utilization (Galinier et al., 1998; Martin-Verstraete et al., 1999). However, during spore revival, it may be activated in the presence of alternative PTS sugars (Rosenberg et al., 2015). In addition, strains producing some HPr mutant proteins on the Ser46 phosphorylation site (both phosphoablative and phosphomimetic mutants) exhibited reduced sporulation efficiency (Rosenberg et al., 2015). These results indicate that the phosphorylation level of HPr is important for spore revival. This is not surprising since optimal carbon utilization needs to take place rapidly upon revival. Therefore, the regulation of spore revival by phosphorylation on Ser and Thr residues is an important mechanism that can be mediated by several types of kinases like STPKs and HprK/P, or even other atypical protein kinases not yet identified. This network of regulations may also be complicated by cross-talk with other phosphorylation systems like BY-kinases (Cousin et al., 2013), two-component systems, or recently identified Arg phosphorylations (Elsholz et al., 2012; Schmidt et al., 2014).

## Conclusion

It is now widely accepted that regulatory Ser/Thr phosphorylation is as present in prokaryotes as in eukaryotes and that enzymes responsible for these modifications are mainly eukaryotic-like Ser/Thr kinases. To date, four of these proteins have been characterized in *B. subtilis* (PrkA, PrkC, PrkD, and YabT) and several examples highlight their regulatory role in cellular physiology during vegetative growth as well as during sporulation. In this mini review, we focused on their regulatory functions in spores and showed that STPKs have relaxed substrate selectivity that confers to the cell a quick way to adapt to the physiological conditions. Taking into account that cross-phosphorylation events occur among STPKs, BY-kinases, and two-component systems, the regulatory network controlling a spore life is highly dynamic and sophisticated. Given the role of spores in many diseases, understanding mechanisms and regulation of spore formation and spore germination has long been a researcher’s interest in order to find a way to get rid of them more efficiently. However, a new interest has now emerged with the use of spores as a tool in biotechnology (Isticato and Ricca, 2014).

## Author Contributions

All authors listed, have made substantial, direct and intellectual contribution to the work, and approved it for publication.

## Conflict of Interest Statement

The authors declare that the research was conducted in the absence of any commercial or financial relationships that could be construed as a potential conflict of interest.
